# Spitz Nevus Arising Within a Black Ink Tattoo: A Case Report of an Extremely Rare Entity

**DOI:** 10.7759/cureus.77180

**Published:** 2025-01-09

**Authors:** Lilyana Petkova, Pavel Pavlov, Dariya L Chivchibashi-Pavlova

**Affiliations:** 1 Pathology, Complex Oncology Center, Shumen, BGR; 2 Clinical Pathology, Complex Oncology Center, Shumen, BGR; 3 Department of Physiology and Pathophysiology, Faculty of Medicine, Medical University of Varna, Varna, BGR

**Keywords:** rare, spitz, spitz nevus, tattoo, tattoo reaction

## Abstract

Tattooing has a long history as a form of self-expression and often signifies social status and group belonging. It has become increasingly popular among young people recently and many young adults are choosing to get tattoos, while older generations tend to have fewer. However, data on tattoo-related complications are limited as this demographic group ages. Adverse reactions can occur during the tattooing process as well as in the aftercare phase, including allergic responses, infections, and neoplasms.

There has been a surge in the reported cases of skin tumors associated with tattoos in recent years, suggesting a growing concern about potential health risks linked to tattooing. The most common tumors associated with tattoos include malignant melanoma, basal cell carcinoma, keratoacanthoma, and squamous cell carcinoma. The rarer types reported include dermatofibrosarcoma protuberans, non-Hodgkin lymphoma, and Spitz nevi arising in red ink tattoos. Spitz nevi hold particular significance as these melanocytic lesions can be misdiagnosed as malignant melanoma despite their benign biological behavior and favorable prognosis. We present a case of Spitz nevus that developed within a black ink tattoo. To our knowledge, this is the fourth recorded instance of a Spitz nevus developing in a tattoo, and the first case specifically associated with black ink.

## Introduction

Body tattooing has been documented since ancient civilizations. Historically, tattooing was associated with social status, rituals, group affiliation, and the identification of slaves and criminals [1]. Over the past century, tattooing has become increasingly popular among younger generations, particularly Generation X and millennials [2,3]. As of 2021, millennials had the highest percentage of individuals with one or more tattoos in the US. In several European countries, including the UK, Germany, France, Spain, and Denmark, 35-48% of people also reported having at least one tattoo [3].

Despite the prevalence of tattoos in these populations, there is still a limited understanding of the associated complications. The "tattooed generation" has not yet aged enough to experience the long-term effects of different inks or laser removal attempts. Tattoo-related adverse skin reactions can be classified into five main categories: infections, inflammatory responses, cosmetic issues, miscellaneous complications, and notably, tumors [4,5]. Before 2005, only 18 cases of skin tumors linked to tattoos were reported. However, the number of cases increased after 2006 with the growing popularity of tattoos. While the first case was published in 1938, a total of 160 cases have been documented to date.

Interestingly, the projected total of these cases for the years 2021-2025 is 86 cases annually, indicating a consistent trend of emerging tumors in tattoos over the past two decades [3]. The most common histological variants of skin tumors that develop in tattoos include malignant melanoma, basal cell carcinoma, keratoacanthoma, and squamous cell carcinoma. Rarer histological types comprise dermatofibrosarcoma protuberans, leiomyosarcoma, B-cell lymphoma, non-Hodgkin lymphoma [3,5], and Spitz nevi [4,6,7]. Spitz nevi hold particular significance as they can be misdiagnosed as malignant melanoma, despite having benign biological behavior and being associated with a favorable prognosis [8].

Spitz nevi are rare melanocytic lesions characterized by large epithelioid cells, spindled cells, or a combination of both, exhibiting maturation with depth [7,8]. They display the typical architecture found in all melanocytic nevi; while compound lesions are the most common, junctional and intradermal variants have also been observed [8]. Spitz nevi are known to be linked to somatic alterations in HRAS and 6q23, as well as to fusions and mutations in tyrosine kinases and serine/threonine kinases such as BRAF, MAP3K8, and MAP2K1 [9,10]. These lesions rarely develop in individuals older than 20 years of age [11]. Clinically, a Spitz nevus often appears as a single, rapidly enlarging dome-shaped lesion or nodule that is pink to brown [8]. There are three documented cases of Spitz nevi occurring within red ink tattoos in the literature [4,6,7]. To our knowledge, no case of Spitz nevi arising within black ink tattoos has been published so far. We discuss a case of a 23-year-old male with a Spitz nevus that developed within a black ink tattoo.

## Case presentation

A 23-year-old male with no family history of malignant melanoma visited a local dermatologist for a lesion within a tattoo on his left forearm, eight months after the tattoo was applied. The patient stated that there had been no previous lesions in that area of skin before the tattooing. An incisional biopsy of the lesion was sent to the Clinical Pathology Department at the Complex Oncology Center in Shumen for pathological evaluation. Macroscopic examination of the buffered formalin-fixed specimen revealed an exophytic, symmetrical, dome-shaped lesion with a base diameter of 0.8 cm and a thickness of 0.5 cm. The sectional profile of the lesion appeared solid and whitish-pink. The lesion was excised with a healthy peripheral clearance margin of 0.2 cm and included subcutaneous tissue measuring 0.2 cm.

**Figure 1:**
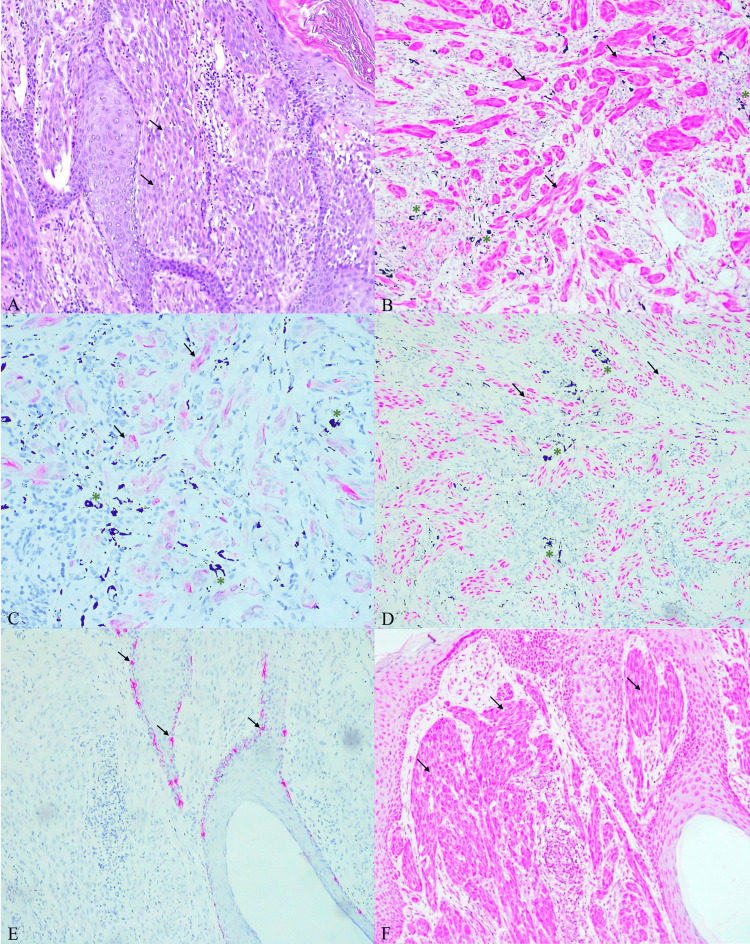
Left forearm excisional biopsy findings A: Hematoxylin and eosin staining showing the proliferation of spindle-shaped, non-pigmented nevomelanocytes in the papillary dermis (arrows). B. Immunohistochemical examination of S100 revealing positive staining in all tumor cells (arrows); pigment granules from black tattoo ink in the macrophages of the stroma between proliferating nevomelanocytes (asterisks). C. Immunohistochemical examination of Melan-A showing positive staining in all tumor cells (arrows) and black ink pigment granules in stromal macrophages (asterisks). D. Immunohistochemical examination of SOX-10 revealing positive expression in all nevomelanocytes (arrows) and pigment granules from black ink in stromal macrophages (asterisks). E. Immunohistochemical examination of HMB-45 showing negative expression in nevomelanocytes, with positive expression in basal melanocytes serving as an internal control (arrows). F. Immunohistochemical examination of BRAF showing positive expression in all nevomelanocytes (arrows). Staining with red pigment (EnVision FLEX HRP Magenta Substrate, Dako Omnis, Agilent Technologies, Santa Clara, CA). Magnification: ×100

Routine histopathological examination showed a dermal, circumscriptive proliferation of spindle-shaped, non-pigmented nevomelanocytes with well-defined eosinophilic cytoplasm, oval vacuolated nuclei, and small, uniform nucleoli (Figure 1A). The nevomelanocytes, accompanied by stromal fibrosclerosis, were arranged in bundles, exhibiting periadnexal growth and maturation with increasing depth of the lesion. Additionally, there was deposition of black pigment from the tattoo ink outside the tumor cells and within stromal macrophages (Figures 1B-1D). Immunohistochemical analysis revealed that all the proliferated nevomelanocytes stained positively for S100 (Figure 1B), Melan-A (Figure 1C), SOX-10 (Figure 1D), and BRAF (Figure 1F), while remaining negative for HMB-45 (Figure 1E). Additionally, we used a red pigment (EnVision FLEX HRP Magenta Substrate, Dako Omnis, Agilent Technologies, Santa Clara, CA) instead of a brown pigment to distinguish it from the brown-colored melanin.

## Discussion

Lesions occurring in tattoos pose a diagnostic challenge for dermatologists and dermatopathologists. Over the years, various reactions to tattooing have been identified. These include local infections, autoimmune diseases such as psoriasis, vitiligo, lupus erythematosus, lichen planus, sarcoidosis, and pyoderma gangrenosum; cosmetic complications; sensory issues like itching and pain; and neoplasms such as malignant melanoma, keratoacanthoma, basal cell carcinoma, and squamous cell carcinoma. Such changes may be related to the concept of an "immunocompromised skin area," which refers to body regions with reduced resistance to disease, similar to the Koebner phenomenon [12].

Spitz lesions are rare and typically do not develop in individuals older than 20 years of age and constitute only about 1% of all nevi in children. The appearance of such lesions in adults should be evaluated by a pathologist specializing in pigmented lesions to rule out malignant melanoma. Clinically, a Spitz nevus may manifest as a solitary, rapidly growing, dome-shaped papule or nodule with a pink-brown color. Less common macroscopic variants include macular, polypoid, pedunculated, and verrucous forms [11,13]. Although manifestations have been described in virtually all body locations - such as the genital mucosa, oral mucosa, tongue, glans penis, penile shaft, subungual area, perianal skin, and soles of the feet - the most frequent occurrences are on the head and neck or limbs. Additionally, while typically solitary, Spitz nevi can also present as multiple lesions. [8].

Based on its primary architecture, the Spitz nevus is classified as composite; however, borderline and intradermal variants can also occur [8,11]. Several histological variants have been documented, including combined, pagetoid, desmoplastic, hyalinized, angiomatoid, halo-associated, pigmented, plexiform, tubular, rosette-like Spitz nevus with Touton-like giant cells, as well as pseudogranulomatous and verrucous Spitz nevi, among others. [11]. Our case was particularly intriguing due to the rarity of Spitz nevi associated with tattoos, specifically those in black ink. The timing and distribution of this lesion raise suspicions that the tattoo may have contributed to its development. However, this could also suggest that the occurrence is merely incidental. To our knowledge, there have been only three reported cases of Spitz nevi in tattoos [4,6,7]. The first case was documented in 2018 when two desmoplastic intradermal Spitz nevi were described in a tattoo created with red ink [4]. In 2024, two articles reported multiple Spitz nevi, again found in a red ink tattoo [6,7].

The 2018 report involved a 28-year-old female who developed two newly appeared intradermal desmoplastic Spitz nevi in the area of red-inked tattooed skin on her left shoulder. The first lesion appeared 8-12 months after the tattooing, while the second lesion appeared three to four years later. Both lesions exhibited similar immunohistochemical expression: positive MITF, S100, SOX-10, and preserved expression of p16 [4]. Similar findings were reported in the two articles from 2024, which described a 30-year-old male with 15 newly emerged Spitz nevi on the skin of his right thigh and left leg within 12 months after tattooing, and a 42-year-old male with multiple Spitz nevi arising from a red ink band tattoo on his right arm. The lesions showed similar immunohistochemical expression as noted in the earlier case from 2018, specifically positive MITF, S100, SOX-10, and preserved expression of p16 [6].

In our patient, a single dome-shaped Spitz nevus arising within a black ink tattoo and whitish-pink in color was observed; it exhibited the typical architecture of Spitz nevi: the proliferation of spindle-shaped non-pigmented nevomelanocytes in the dermis with maturation in depth. To confirm the melanocytic nature of the lesion, we employed an immunohistochemical panel that included S100, Melan A, HMB-45, SOX-10, and BRAF. We included the BRAF stain in the immunohistochemical panel based on an article published by Fullen et al., which identified mutations in BRAF in some spitzoid lesions, particularly in Spitz nevi with atypical histological features [10].

## Conclusions

The diagnostic challenges posed by lesions arising in tattoos underscore the complexities encountered by dermatologists and dermatopathologists. As tattooing becomes increasingly mainstream, healthcare providers should remain vigilant regarding potential complications and approach any unusual lesions with a high degree of suspicion. Our case, along with three previously published reports of tattoo-associated Spitz nevi, suggests a possible biological response to tattoo ink that warrants further investigation. Continued research and documentation of such cases will not only enhance our understanding of the relationship between tattoos and skin lesions but also illuminate crucial yet unexplored pathways for reactive nevogenesis. Fostering greater awareness of these issues can ultimately improve patient care and outcomes.

## References

[1] Laumann AE (2010). History and epidemiology of tattoos and piercings. Legislations in the United States. Dermatologic Complications with Body Art.

[2] Kluger N (2015). Epidemiology of tattoos in industrialized countries. Curr Probl Dermatol.

[3] Lebhar J, Jacobs J, Rundle C, Kaplan SJ, Mosca PJ (2024). Skin cancers arising within tattoos: a systematic review. JAAD Int.

[4] Saunders BD, Nguyen M, Joo JS, Konia TH, Tartar DM (2018). Desmoplastic intradermal spitz nevi arising within red tattoo ink. Dermatol Online J.

[5] Huisman S, van der Bent SA, Maijer KI, Tio DC, Rustemeyer T (2020). Cutaneous non-allergic complications in tattoos: an overview of the literature. Presse Med.

[6] Latoni DI, Foreman RK, Lavigne K, Busam KJ, Tsao H (2024). Multiple de novo spitzoid nevi arising within a specific red tattoo ink. JAAD Case Rep.

[7] Bettolini L, Bighetti S, Rovaris S, Ghini I, Calzavara-Pinton P, Maione V (2024). Spitz nevus puzzle: red ink tattoo unveiling rare plexiform variants. Australas J Dermatol.

[8] Calonje E, Brenn T, Lazar AJ, Billings SD (2019). J McKee's Pathology of the Skin, 5th Edition. J McKee's Pathology of the Skin.

[9] Hagstrom M, Fumero-Velázquez M, Dhillon S, Olivares S, Gerami P (2023). An update on genomic aberrations in Spitz naevi and tumours. Pathology.

[10] Fullen DR, Poynter JN, Lowe L (2006). BRAF and NRAS mutations in spitzoid melanocytic lesions. Mod Pathol.

[11] Casso EM, Grin-Jorgensen CM, Grant-Kels JM (1992). Spitz nevi. J Am Acad Dermatol.

[12] Caccavale S, Kannangara AP, Ruocco E (2016). The immunocompromised cutaneous district and the necessity of a new classification of its disparate causes. Indian J Dermatol Venereol Leprol.

[13] Cho SB, Kim HS, Jang H (2009). A pedunculated hyalinizing Spitz nevus on the penile shaft. Int J Dermatol.

